# Department of Dermatology

**Published:** 1893-02

**Authors:** Isadore Dyer

**Affiliations:** Tulane University, New Orleans, La.


### DEPARTMENT OF DERMATOLOGY.

---

Dermatological Notes by Isadore Dyer, M. D., Tulane University, New Orleans, La.

---

### MILIARIA CRYSTALLINA.

In his statistics of eight thousand cases of skin diseases, Dr. Bulkley reports one of miliaria. Doubt still exits as to the identity of miliaria and sudamina. The concensus of opinion of the American and English dermatologist favors a common classification. Some (Drs. Bulkley, Anderson, Tilbury Fox and Jamison, notably) consider miliaria and sudamina identical. Others (Van Harlingen, Taylor, Ratcliffe, Crocker, with Brocq and Guibot of Paris) describe miliaria, and relegate sudamina to that type which they recognize as miliaria crystallina. Still others (Duhring, Shoemaker, Stellwagen and Morrow) differentiate between miliaria and sudamina, using the term miliaria crystallina as a synonym of sudamina, but as having no connection with other types of miliaria.

Sudamina is purely a process in evidence of a morbid condition of the sweat glands. It is associated with some easily recognized constitutional derangement, but may occur independent of any constitutional evidence whatsoever. It is attended with, or follows a high state of hyperidrosis, and seems distinctly a functional disturbance. Its lesions are translucent, whitish vesicles, pin point or pin head sized, with indiscriminate localization; but preferring the neck, chest, abdomen and rest of trunk. The lesions are discrete, and remain so throughout the disease, never becoming confluent. even though they become quite closely aggregated.

Dr. Moritz Kapozi, of Vienna, discusses sudamina as a disease distinct from miliaria. Sudamina he considers a disease of the vesicular type. He divides miliaria into three varieties, with distinct prodromic and concurrent srmptoms, all or any of which may be present. These three varieties are: 1° miliaria rubra; 2° miliaria albida; 3° miliaria crystallina. M. Crystallina, according to Kaposi, has the evidences of a veritable exanthem. The vesicles of M. Crystallina are generally millet seed in size. They are clear like water, pale, resembling dew drops. They are often more sensible to touch than to sight. These vesicles persist several days or several weeks, depending upon the conditions causing them. Their contents have a reaction faintly alkaline, never acid. The contents never change, never become purulent, always remain in an eruption resembling dew drops.

Dr. E. Geber, of Klausenberg, in Ziemmsen's Handbook of Diseases of the Skin, considers miliaria a definite disease of sudoral type, but distinct from sudamina. Sudamina, he holds inflammatory at times, the fluid of its lesions capable of becoming cloudy or purulent. Miliaria crystallina he recognizes as a distinct variety of miliaria, with constitutional symptoms, which, with the eruption, constitutes a disease *sui generis*. Personally, I cannot presume to offer any classification, and with such differing opinions as these references indicate, it would be hard to define miliaria crystallina or sudamina. Recently a case presented itself at my clinic in the out door department of Charity hospital, and its clear cut clinical history makes it an interesting case in point to report. Lydia P., girl of thirteen, colored; plethoric type. Attends school; is a hard student; exercise limited.

Three months previous grew stupid. Malaise, occasional chill. Headaches frequent. Bowels move each day. Has never menstruated. Eruptions appeared two months before day of visit. Eruption has not changed since first appearance. No pain. No distress whatever at the site of eruption. Stupidity of child has gradually increased since eruption appeared. Child sleeps most of the time. Still attends school, however.

These points were obtained from the mother, the child being too stupid and drowsy to answer any question intelligently. The eruption was limited to the right cheek, upper lip and chest. At each of these points there was a patch of closely aggregated vesicles, pin-head in size and resembling dew-drops. There was no apparent inflammation at the site of the patches, and the eruption looked as if it might be brushed or wiped away. Each patch was sharply defined. The patch on the right cheek lay in a slanting or diagonal direction across the cheek, appearing to follow the course of the superior maxillary branch of the trifacial nerve. This conception was further suggested by the similarly grouped vesicles along the upper lip.

The mother was instructed to keep the child from school, and to promote exercise.

A desiccant lotion was prescribed; while internally two grains of antipyrin, three drops of Fowler's solution, in cinnamon water, was given after each meal.

Three days later the child returned, with no eruption on the chest and the two patches on the face drying up.

Exactly one week after the first visit the child returned with no evidence of eruption, bright and cheerful, and, as the mother expressed it, herself again.

I have called this case one of miliaria crystallina, because the prodeomic and concomitant manifestations appeared to be directly connected with the eruption. These conditions disappeared at the same time as the eruption, and both seemed to be dependent one on the other. The readiness with which the antipyrin and arsenic seemed to control the conditions, indicates an immediate vasomotor connection, and adds clinical evidence of a sudoral influence which the pathological examinations of such lesions tend to prove.

